# Injury-related mortality in South Africa: a retrospective descriptive study of postmortem investigations

**DOI:** 10.2471/BLT.14.145771

**Published:** 2015-03-13

**Authors:** Richard Matzopoulos, Megan Prinsloo, Victoria Pillay-van Wyk, Nomonde Gwebushe, Shanaaz Mathews, Lorna J Martin, Ria Laubscher, Naeemah Abrahams, William Msemburi, Carl Lombard, Debbie Bradshaw

**Affiliations:** a Burden of Disease Research Unit, South African Medical Research Council, PO Box 19070, Tygerberg, 7505, Cape Town, South Africa.; b Biostatistics Unit, South African Medical Research Council, Cape Town, South Africa.; c Gender and Health Research Unit, South African Medical Research Council, Cape Town, South Africa.; d Division of Forensic Medicine and Toxicology, University of Cape Town, Cape Town, South Africa.

## Abstract

**Objective:**

To investigate injury-related mortality in South Africa using a nationally representative sample and compare the results with previous estimates.

**Methods:**

We conducted a retrospective descriptive study of medico-legal postmortem investigation data from mortuaries using a multistage random sample, stratified by urban and non-urban areas and mortuary size. We calculated age-specific and age-standardized mortality rates for external causes of death.

**Findings:**

Postmortem reports revealed 52 493 injury-related deaths in 2009 (95% confidence interval, CI: 46 930–58 057). Almost half (25 499) were intentionally inflicted. Age-standardized mortality rates per 100 000 population were as follows: all injuries: 109.0 (95% CI: 97.1–121.0); homicide 38.4 (95% CI: 33.8–43.0; suicide 13.4 (95% CI: 11.6–15.2) and road-traffic injury 36.1 (95% CI: 30.9–41.3). Using postmortem reports, we found more than three times as many deaths from homicide and road-traffic injury than had been recorded by vital registration for this period. The homicide rate was similar to the estimate for South Africa from a global analysis, but road-traffic and suicide rates were almost fourfold higher.

**Conclusion:**

This is the first nationally representative sample of injury-related mortality in South Africa. It provides more accurate estimates and cause-specific profiles that are not available from other sources.

## Résumé

**Objectif:**

Enquêter sur la mortalité par traumatisme en Afrique du Sud à partir d'un échantillon nationalement représentatif et comparer les résultats avec les estimations antérieures.

**Méthodes:**

Nous avons réalisé une étude descriptive rétrospective des données de rapports médico-légaux obtenus auprès de morgues, en utilisant une technique d'échantillonnage aléatoire à plusieurs degrés, stratifié en fonction des critères suivants : zone urbaine, zone non urbaine et taille de la morgue. Nous avons calculé les taux de mortalité par âge et les taux de mortalité standardisés selon l’âge, pour les différentes causes externes de décès.

**Résultats:**

Les dossiers d'autopsie font état de 52.493 décès par traumatisme en 2009 (intervalle de confiance de 95 %, IC : 46.930–58.057). Quasiment la moitié (25.499 décès) correspond à des blessures infligées intentionnellement. Taux de mortalité standardisé selon l’âge pour 100.000 personnes : Tous types de blessures : 109,0 (IC 95 % : 97,1–121,0) ; Homicides 38,4 (IC 95% : 33,8-43) ; Suicides 13,4 (IC 95% : 11,6-15,2) ; Blessures liées à des accidents de transport 36,1 (IC 95% : 30,9–41,3). Les rapports d'autopsie indiquent un nombre de décès par homicide et accident de la route plus de trois fois supérieur aux chiffres recensés dans les registres d'état civil pour la même période. Le taux d'homicide a confirmé les estimations préalablement faites pour l'Afrique du Sud dans une analyse internationale. En revanche, les taux correspondant aux accidents de la route et aux suicides se sont avérés près de quatre fois plus élevés.

**Conclusion:**

Il s'agit du tout premier échantillon nationalement représentatif pour la mortalité par traumatisme en Afrique du Sud. Cette étude offre des estimations plus précises et des profils détaillés par causes, qui ne sont disponibles dans aucune autre source.

## Resumen

**Objetivo:**

Investigar la mortalidad relacionada con los traumatismos en Sudáfrica utilizando una muestra representativa a nivel nacional y comparar los resultados con estimaciones anteriores.

**Métodos:**

Se llevó a cabo un estudio retrospectivo y descriptivo de datos médico-legales de investigaciones post mortem utilizando una muestra aleatoria en varias etapas, estratificado por zonas urbanas y zonas no urbanas y el tamaño de la morgue. Se calcularon las tasas de mortalidad específicas y normalizadas por edades de causas de muerte externas.

**Resultados:**

Los informes post mortem revelaron 52.493 muertes relacionadas con traumatismos en 2009 (intervalo de confianza, IC, del 95%: 46.930–58.057). Casi la mitad (25.499) fueron infligidas intencionadamente. Las tasas de mortalidad normalizadas por edades por 100.000 habitantes fueron las siguientes: todos los traumatismos: 109,0 (IC del 95%: 97,1–121,0); homicidio 38,4 (IC del 95%: 33,8–43); suicidio 13,4 (IC del 95%: 11,6–15,2) y traumatismos relacionados con el transporte 36,1 (IC del 95%: 30,9–41,3). Utilizando informes post mortem, se observaron tres veces más casos de muertes por homicidio y traumatismos por accidentes de tráfico que la cantidad registrada en el registro civil para este periodo. La tasa de homicidio fue similar a la estimación para Sudáfrica desde una perspectiva global, pero las tasas de accidentes de tráfico y suicidios fueron casi cuatro veces superiores.

**Conclusión:**

Esta es la primera muestra representativa a nivel nacional de mortalidad relacionada con los traumatismos en Sudáfrica. Proporciona estimaciones más precisas y perfiles por causas específicas que no pueden obtenerse de otras fuentes.

## ملخص

**الغرض:**

استقصاء الوفيات المرتبطة بالإصابات في جنوب أفريقيا باستخدام عينة ممثلة على المستوى الوطني ومقارنة النتائج بالتقديرات السابقة. ****

**الطريقة:**

أجرينا دراسة وصفية استعادية لبيانات الاستقصاء التشريحية على مستوى الطب الشرعي، من مستودعات الجثث باستخدام عينة عشوائية متعددة المراحل، مقسمة تبعاً للمناطق الحضرية وغير الحضرية وحجم مستودع الجثث. وقمنا بحساب معدلات الوفيات النوعية للعمر، والمقيّسة بالعمر للأسباب الخارجية للوفاة.

**النتائج:**

أظهرت التقارير التشريحية أن 52493 حالة وفاة مرتبطة بالإصابات في 2009 (بنسبة أرجحية مقدارها 95%، نسبة الأرجحية: (46930 إلى 58057). وأُصيب النصف تقريباً (25499) عن عمد. وجاءت معدلات الوفيات المقيّسة بالعمر لكل 100000 نسمة كما يلي: جميع الإصابات: 109.0 (بنسبة أرجحية مقدارها 95%: 97.1 إلى 121.0)؛ والقتل 38.4 (بنسبة أرجحية مقدارها 95%: 33.8 إلى 43؛ والانتحار 13.4 (بنسبة أرجحية مقدارها 95%: 11.6 إلى 15.2) والإصابات المرتبطة بالنقل 36.1 (بنسبة أرجحية مقدارها %95: (30.9 إلى 41.3). باستخدام التقارير التشريحية، وجدنا أن الوفيات الناتجة عن القتل والإصابات المرورية على الطرق كانت أكثر من التي تم تسجيلها في السجلات المدنية في هذه الفترة بأكثر من ثلاثة أمثال. كان معدل القتل مشابهاً للتقدير الخاص بجنوب أفريقيا من التحليل العالمي، ولكن ازدادت معدلات الحوادث المرورية على الطرق والانتحار بأربعة أمثال تقريباً.

**الاستنتاج:**

هذه أول عينة ممثلة على المستوى الوطني للوفيات المرتبطة بالإصابات في جنوب أفريقيا. وتوفر العينة تقديرات أكثر دقة ومرتسمات خاصة بالأسباب غير متاحة في مصادر أخرى.

## 摘要

**目的:**

旨在采用具有全国代表性的样本调查南非境内与伤害有关的死亡率，并与之前的评估进行结果对比。

**方法:**

我们按照城市和非城市区域以及停尸房的大小来分层，采用多级随机抽样法从停尸房处获得法医学尸检调查数据，从而开展了一项回溯式的描述性研究。我们针对外部原因致死的情况计算了特定年龄和标准化年龄的死亡率。

**结果:**

尸检报告显示 2009 年发生 52 493 起与伤害有关的死亡事件（95% 置信区间，CI：46 930–58 057）。其中几乎一半 (25 499) 为故意造成。每 100 000 人口的标准化年龄死亡率如下：所有受伤情况：109.0 (95% CI:97.1–121.0)；凶杀 38.4 (95% CI:33.8–43)；自杀 13.4 (95% CI:11.6–15.2)，以及因交通事故而受伤 36.1 (95% CI:30.9–41.3)。通过尸检报告，我们发现这个时期因凶杀和道路交通事故而死亡的事件是人口动态登记处所记录的三倍以上。凶杀率与全球分析中对南非的评估相似，但是道路交通事故死亡率和自杀率几乎高出四倍。

**结论:**

这是第一次采用具有全国代表性的样本调查南非境内与伤害有关的死亡率，从而提供了更加精确的评估和描述具体原因的文件，弥补了其他资料来源在这方面的不足。

## Резюме

**Цель:**

Исследование смертности от травм в Южной Африке с помощью национально-репрезентативной выборки и сравнение результатов с предыдущими оценками.

**Методы:**

Мы провели ретроспективное описательное исследование данных судебно-медицинских вскрытий, полученных из моргов, с помощью многоступенчатой случайной выборки с разбивкой по городским и негородским районам и размеру морга. Мы определили возрастозависимый уровень смертности и уровень смертности, стандартизированный по возрасту, для случаев смерти, наступившей от внешних причин.

**Результаты:**

Согласно данным посмертных эпикризов в 2009 году было зарегистрировано 52 493 смерти от травм (доверительный интервал 95 %, ДИ: 46 930–58 057). Почти половина травм (25 499) была нанесена умышленно. Показатели смертности, стандартизированные по возрасту, на 100 000 населения распределились следующим образом. Все виды травм: 109,0 (95 % ДИ: 97,1–121,0); убийство: 38,4 (95 % ДИ: 33,8–43); самоубийство: 13,4 (95 % ДИ: 11,6–15,2); травмы, связанные с транспортом: 36,1 (95 % ДИ: 30,9–41,3). В ходе исследования посмертных эпикризов мы обнаружили, что количество смертей в результате убийств и дорожно-транспортного травматизма более чем в три раза превышало показатель, зарегистрированный в реестре актов гражданского состояния за этот период. Данные по количеству убийств были сопоставимы с данными для Южной Африки, полученными в результате глобального анализа, а количество смертей от дорожно-транспортного травматизма и самоубийств оказалось почти в четыре раза выше.

**Вывод:**

Это первая национально-репрезентативная выборка смертности от травм в Южной Африке. Она предоставляет более точную оценку и сведения в зависимости от причинного фактора, которые невозможно получить из других источников.

## Introduction

In South Africa in the year 2000, injury-related mortality accounted for 12% of deaths and 16% of years of life lost.^1^ This was primarily due to high mortality rates from road-traffic injury and homicide, which were approximately twice and eight times the global average, respectively.^1^^,^^2^

A previous South African national study of the burden of injury-related mortality used triangulation and modelling techniques^2^ to overcome deficiencies in vital registration data and national statistics, such as underreporting^3^^,^^4^ and the urban bias of national injury mortality surveillance.^5^^,^^6^ These surveillance data are no longer suitable for burden of disease modelling. They are not nationally representative, since they are only available for two of nine provinces, and use mortuary registers rather than postmortem reports.^7^ For deaths of undetermined cause, mortuary registers fail to differentiate routinely between deaths from natural or external causes and, for external-cause deaths, between accidental and deliberate events.

Under the Inquests Act of 1959,^8^ postmortem investigations are a statutory requirement for all deaths that are not clearly from natural causes. This is a potentially useful alternative source of data on injury-related mortality. Here, we use postmortem records to provide a more accurate cause-specific profile of injury-related mortality in South Africa for the year 2009. This enables comparison with data from several sources including official statistics, the national survey of female homicides^9^ and global burden of disease estimates. The study was commissioned by the South African Medical Research Council as part of its second national burden of disease study.^10^

## Methods

We conducted a retrospective descriptive study, using routine data collected through postmortem investigations during 2009. Data were obtained from postmortem reports and ancillary documentation, including police reports and hospital records. A multistage stratified cluster sample was drawn for eight provinces, using mortuaries as the primary sampling unit. A sampling frame of 57 274 postmortem reports from 106 mortuaries was used to draw a representative sample stratified by metro and non-metro area and mortuary size (stratified as less than 500, 501–1500, and more than 1500 cases). Forty-five mortuaries were selected with an expected sample of 22 733 records. All records for the Western Cape were obtained from the Provincial Injury Mortality Surveillance System^11^ to complete the national sample. We assessed whether each death was from natural, external or undetermined cause. Field workers recorded the date of death, circumstances of death and the apparent manner of death (homicide, suicide, transport-related, or other unintentional or undetermined intent) consistent with the 10th revision of the International Statistical Classification of Diseases and Related Health Problems*,* 2007 (ICD-10; Table 1). 

**Table 1:** Categories included in the injury-related mortality survey and corresponding ICD-10 codes, South Africa, 2009

Cause of injury	ICD-10 code
Homicide	X85–X99, Y00–Y09
Suicide	X60–X84
Transport injuries	V00–V99
Road traffic injuries	V00–V04,V06, V09–V80, V82–V85, V87, V89
Other transport injuries	V05, V81, V86, V88, V90–V99
Poisonings	X40–X49, X67–X69
Falls	W00–W19
Fires, heat and hot substances	X00–X19
Drowning	V90, V92, W65–W70, W73, W74
Mining accidents	W77, Y37
Other threats to breathing	W75–W84
Mechanical forces	W24–W34, W45–W46
Exposure to natural forces	X30–X39
Adverse effects of medical and surgical treatment	Y39–Y66, Y68–Y84, Y88
Animal contact Other unintentional injuries	W53–W59, X20–X27, X29 W20–W23, W35–W44, W49–W52, W60, W64, W85–W94, W99, X28, X50–X59, Y38
Unspecified or not listed	Y09, Y10-Y34, Y36, Y85-Y87, Y89

ICD-10: International Statistical Classification of Diseases, 10^th^ revision (2007).

We excluded deaths from natural causes, fetal deaths and deaths that occurred outside South Africa. To account for the selection probabilities of mortuaries within survey strata, we applied analysis weights. Cases with unknown-age were proportionally redistributed to all other ages using a scaling factor. Age-standardized mortality rates were calculated for manner of death by age, sex, race, metro and non-metro area using alternate mid-year population estimates^12^ and the World Health Organization’s (WHO) world standard population.^13^

We recruited field workers and tested them for their ability to extract data from records. Field workers used a mobile phone based questionnaire to collect demographic information from the postmortem report, including age, sex and race of the deceased. Postmortem and police reports categorize individuals by the races black, coloured, Asian and white, and we kept those categories when conducting the study. We also recorded whether each death was related to a legal intervention, occurred in custody or if there was evidence of sexual assault. The mortuary death register number and the death notification number were collected as identifiers for follow-up to resolve data capture errors. The data captured on the mobile phone questionnaire application (Mobenzi Researcher, Cape Town, South Africa) were submitted to a central web-based platform. The project manager and biostatistician conducted quality checks while data were collected and resolved any data quality problems with the national level coordinator. Interobserver reliability was tested by two fieldworkers collecting data independently from the same folder on the same day for 5% of the sample. Reliability was high for cause of death (*Κ* = 0.86; 95% confidence interval, CI: 0.84–0.88), age (*Κ* = 0.95; 95% CI: 0.93–0.98) and sex (*Κ* = 0.94; 95% CI: 0.92–0. 97). Further details are available from corresponding author.

### Ethics

The South African Medical Research Council’s Health Research Ethics Committee approved the study.

## Results

**Fig. 1:**
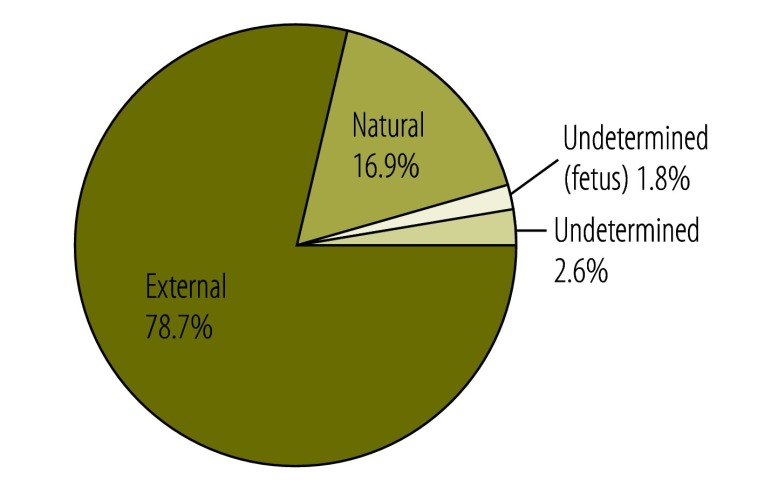
Cause of death recorded by mortuaries, South Africa, 2009

Note: Weighted analysis, *n* = 66 693.

A total of 22 583 cases were drawn from the eight provinces – more than 99% of the expected total of 22 733. The discrepancy arose from invalid entries that had been included in the sampling frame – such as deaths that occurred before 2009 – and a small number of cases not recorded in mortuary registers or lacking records. A further 9418 cases were appended from the Western Cape database, providing a total unweighted data set of 32 001 records. After the application of sampling weights it was estimated that a total of 66 693 (95% CI: 60 356–73 030) deaths were processed by the forensic pathology service in 2009, of which 52 493 (95% CI: 46 930–58 057), or 78.7%, were from external causes (Fig. 1).

The age-standardized mortality rate from all external causes in South Africa in 2009 was 109.0 per 100 000 population (95% CI: 97.1–121.0). The mortality rate among males (181.0; 95% CI: 161.3–200.7) was significantly higher than for females (42.7; 95% CI: 37.1–48.4), equivalent to 4.2 male deaths per female death (Table 2).

**Table 2:** Apparent manner and major external causes of mortality, South Africa, 2009

Apparent manner and external causes of death	Females^a^			Males^a^			Total^a^		RR
	No. (95% CI)	Mortality rate per 100 000 population (95%CI)^b,c^		No. (95% CI)	Mortality rate per 100 000 population (95%CI)^b,c^		Male / female	Mortality rate per 100 000 population (95%CI)^b,c^	
**All injuries (V01–Y34)**	10 541 (9 306–11 777)	42.7 (37.1– 48.4)		41 807 (37 431–46 183)	181.0 (161.3–200.7)		52 493 (46 930–58 057)	109 (97.1–121.0)	4.2
**Intentional injuries (X60–Y09)**	3 894 (3 442–4 345)	15.7 (13.4–18.1)		21 552 (19 248–23 856)	90.6 (80.1–101.1)		25 499 (22 769–28 229)	51.8 (46.0–57.7)	5.8
**Homicide (X85–Y09)**	2 740 (2 440–3 041)	11.3 (9.5–13.0)		16 245 (14 339–18 151)	67.4 (58.9–75.8)		19 028 (16 852–21 204)	38.4 (33.8–43.0)	6.0
Sharp force/stabbing (X99)	823 (724–923)	3.4 (2.7–4.1)		7112 (6 174–8 050)	28.1 (24.0–32.1)		7 951 (6 945–8 957)	15.4 (13.3–17.6)	8.3
Firearm injuries (X93–X95)	611 (539–684)	2.5 (1.9–3.1)		4895 (4 381–5 408)	20.5 (18.0–23.1)		5 513 (4 937–6 090)	11.2 (9.9–12.6)	8.2
Blunt force (Y00)	735 (628–843)	3.1 (2.3–3.8)		3595 (3 070–4 120)	15.4 (12.7–18.2)		4 336 (3 729–4 942)	9.0 (7.5–10.4)	5.0
Strangulation/threats to breathing (X91)	315 (261–368)	1.2 (0.8–1.6)		222 (179–266)	1.0 (0.5–1.5)		538 (463–614)	1.1 (0.7–1.4)	0.8
Fire/burns (X97–X98)	79 (51–107)	0.4 (0.1–0.7)		121 (78–165)	0.6 (0.2–1.0)		203 (137–269)	0.5 (0.2–0.8)	1.5
Poisoning/ingestion (X85)	72 (32–112)	0.3 (0.1–0.5)		112 (59–166)	0.5 (0.1–1.0)		184 (94–275)	0.4 (0.1–0.6)	1.7
Other^d^	87 (65–109)	0.3 (0.2–0.5)		142 (95–188)	0.6 (0.3–1.0)		240 (180–299)	0.5 (0.3–0.7)	2.0
Unknown (Y09)	17 (8–27)	0.1 (0–0.1)		45 (22–69)	0.2 (0.1–0.3)		63 (37–88)	0.1 (0.1–0.2)	2.0
**Suicide (X60–X84)**	1 153 (976–1 331)	4.5 (3.5–5.4)		5 307 (4 717–5 898)	23.2 (19.9–26.5)		6 471 (5 753–7 189)	13.4 (11.6–15.2)	5.2
Hanging (X70)	488 (410–567)	1.9 (1.4–2.3)		3 651 (3 170–4 131)	15.5 (12.8–18.2)		4 148 (3 613–4 683)	8.4 (7.0–9.8)	8.2
Poisoning/ingestion (X60–X65)	463 (351–575)	1.8 (1.2–2.3)		636 (531–741)	2.7 (2.1–3.3)		1 099 (910–1 288)	2.2 (1.7–2.7)	1.5
Gunshot injuries (X72–X74)	93 (64–123)	0.4 (0.2–0.6)		686 (575–798)	3.4 (2.5–4.2)		780 (653–907)	1.8 (1.3–2.2)	8.5
Poisoning/gassing (X66–X69)	38 (24–51)	0.2 (0–0.3)		114 (87–142)	0.5 (0.4–0.7)		152 (117–187)	0.3 (0.2–0.5)	2.5
Jump from height (X80)	37 (22–51)	0.2 (0–0.3)		47 (25–69)	0.2 (0.1–0.4)		83 (50–117)	0.2 (0.1–0.3)	1.0
Other^e^	30 (19–42)	0.1 (0–0.2)		168 (133–203)	0.8 (0.5–1.0)		198 (161–236)	0.4 (0.3–0.6)	8.0
Unknown (X84)	4 (1–8)	0 (0–0)		5 (2–9)	0 (0–0)		9 (5–13)	0 (0–0)	2.3
**Unintentional injuries (V00–X59)**	6 186 (5 373–6 999)	25.2 (21.2–29.1)		18 637 (16 444–20 830)	82.9 (72.5–93.3)		24 895 (21 924–27 867)	52.7 (46.0–59.4)	3.3
**Transport (V00–V99)**	4 229 (3 575–4 882)	16.1 (13.2–19.0)		13 486 (11 741–15 231)	58.2 (50.1–66.3)		17 742 (15 366–20 118)	36.1 (31.1–41.2)	3.6
Road traffic pedestrian injuries (V00–V04)	1 299 (1 149–1 448)	5.3 (4.2–6.4)		4 290 (3 855–4 726)	19.2 (16.7–21.8)		5 604 (5 027–6 181)	11.9 (10.4–13.5)	3.6
Motor vehicle passenger injuries (V40–V79 [.1;.6])	1 760 (1 383–2 138)	7.1 (5.2–9.0)		2 802 (2 281–3 323)	11.6 (9.1–14.2)		4 572 (3 679–5 464)	9.3 (7.3–11.4)	1.6
Motor vehicle driver injuries (V40–V79 [.0;.5])	313 (251–376)	1.4 (0.9–1.8)		2 891 (2 386–3 397)	13.3 (10.7–16.0)		3 205 (2 655–3 755)	7.0 (5.7–8.4)	9.5
Road traffic, unspecified type (V40–V79 [.2;.3;.4])	727 (409–1 045)	2.8 (1.5–4.0)		2 365 (1 569–3 162)	10.2 (6.5–13.9)		3 093 (1984–4 202)	6.3 (4.0–8.6)	3.6
Rail pedestrian injuries (V05)	72 (45–98)	0.3 (0.1–0.5)		371 (294–448)	1.6 (1.2–2.1)		443 (350–537)	0.9 (0.7–1.2)	5.3
Other^f^	57 (39–74)	0.2 (0.1–0.3)		720 (580–860)	3.2 (2.2–4.1)		777 (630–925)	1.6 (1.2–2.1)	16.0
Unknown (V99)	1 (1–1)	0 (0–0)		47 (7–86)	0.2 (0–0.4)		48 (9–87)	0.1 (0–0.2)	43.7
**All road-traffic injuries (V00–V89)**	4135 (3 492–4 778)	16.8 (13.7–20)		12 942 (11 245–14 639)	57.2 (49–65.3)		17 103 (14 781–19 425)	36.1 (30.9–41.3)	3.4
**Other unintentional injuries (W00–X59)**	1 958 (1 727–2 188)	6.4 (5.3–7.6)		5151 (4 608–5 693)	21.4 (18.5–24.3)		7153 (6 411–7 895)	13.5 (11.8–15.2)	3.3
Fire/burns (X00–X19)	720 (613–828)	2.9 (2.2–3.6)		1 239 (1 108–1 371)	5.6 (4.5–6.7)		1 973 (1 751–2 195)	4.2 (3.5–4.9)	1.9
Drowning (W65–W74)	307 (243–371)	1.2 (0.7–1.6)		1 376 (1 159–1 593)	5.7 (4.4–7.0)		1 690 (1 430–1950)	3.3 (2.6–4.1)	4.8
Falls (W00–W19)	154 (120–187)	0.1 (0.1–0.2)		538 (442–634)	2.9 (2.1–3.7)		697 (572–823)	1.7 (1.2–2.1)	29.0
Surgical and medical complications (Y40–Y84)	216 (168–264)	0.9 (0.6–1.2)		182 (134–230)	1.0 (0.6–1.3)		402 (312–492)	0.9 (0.7–1.2)	1.1
Poisoning/ingestion (X40–X43)	104 (76–132)	0.0 (0–0.1)		231 (164–298)	1.0 (0.5–1.4)		337 (255–419)	0.7 (0.4–0.9)	38.8
Electrocution (W85–W99)	66 (45–86)	0.2 (0.1–0.4)		200 (166–234)	0.8 (0.5–1.1)		267 (228–305)	0.5 (0.3–0.7)	4.0
Lightning (X33)	60 (32–88)	0.2 (0–0.5)		198 (150–246)	0.9 (0.4–1.4)		258 (186–330)	0.5 (0.3–0.8)	4.5
Suffocation/threats to breathing (W75–W84)	53 (35–72)	0.0 (0–0)		152 (111–192)	0.5 (0.2–0.8)		205 (154–256)	0.3 (0.1–0.5)	113.4
Other^g^	139 (106–172)	0.2 (0.1–0.3)		675 (557–792)	3.0 (2.1–3.9)		819 (676–962)	1.8 (1.3–2.2)	15.0
Unknown (X59)	109 (101–118)	0.5 (0.4–0.6)		187 (166–207)	1.0 (0.8–1.2)		303 (278–328)	0.7 (0.6–0.8)	2.0
**Undetermined intent (Y09–Y34, Y36, Y85–Y87, Y89)**	461 (340–582)	1.8 (1.2–2.5)		1 618 (1 269–1 967)	7.6 (5.7–9.5)		2 099 (1 643–2 554)	4.6 (3.5–5.7)	4.2
Poisoning/ingestion (Y10–Y15)	121 (86–156)	0.5 (0.2–0.7)		293 (217–368)	1.3 (0.8–1.8)		417 (314–520)	0.9 (0.6–1.2)	2.6
Fire/burns (Y25–Y27)	101 (61–141)	0.4 (0.2–0.6)		179 (96–262)	0.9 (0.3–1.4)		283 (164–402)	0.6 (0.3–0.9)	2.3
Other^h^	154 (106–202)	0.5 (0.2–0.7)		728 (595–862)	3.3 (2.4–4.2)		882 (711–1 054)	1.8 (1.3–2.3)	6.6
Unknown (Y34)	85 (59–111)	0.3 (0.1–0.5)		418 (303–533)	1.9 (1.1–2.7)		517 (391–643)	1.1 (0.7–1.5)	6.3

CI: confidence interval; RR: rate ratio.

^a^ For females: unweighted *n* = 4831 and weighted  *n* = 10 541. For males: unweighted *n* = 19 283 and weighted *n* = 41 807. For total: unweighted *n* =  24 197 and weighted  *n* = 52 493.

^b^ The numbers for total deaths exceed the sum of deaths by sex due to 145 deaths in which sex could not be determined.

^c^ Age-standardized to the WHO world standard population.

^d^ Include abandoned babies (Y06: 69 deaths), pushing (Y01: 27 deaths), drowning (X92: 26 deaths), poisoning/gassing (X86–X90: 25 deaths).

^e^ Included sharp force injuries (X78: 62 deaths), fire/burns (X75–X77: 52 deaths) and railway pedestrian injuries (X81–X82: 48 deaths).

^f^ Include fatal injuries to motor-cycle riders (V20-V29[.0;.4]: 377 deaths), bicycle riders (V10–V19[.0;.4]: 234 deaths), railway passengers (V81: 70 deaths), aviation (V95–V97: 40 deaths) and motor-cycle passengers (V20–V29[.1;.5]: 19 deaths).

^g^ Include blunt-force injuries (W20–W22: 29 deaths), crushing (W23: 186 deaths), poisoning/gassing (X44–X49: 130 deaths), environmental exposure (X30–X32: 121 deaths), animal contact (W53–W59; X20–X27; X29: 111 deaths), circumcision (Y62–Y66: 83 deaths), sharp force injuries (W25–W27: 48 deaths), gunshot injuries (W32–W34: 48 deaths), injuries from machinery (W28–W31: 26 deaths) and explosive blasts (W35–W40: 19 deaths).

^h^ Include blunt force (Y29: 273 deaths), drowning (Y21: 125 deaths), gunshot injuries (Y22–Y24: 87 deaths), poisoning/gassing (Y16–Y19: 85 deaths), falls (Y30–Y31: 69 deaths), hanging (Y20: 66 deaths), strangled (Y20: 37 deaths), surgical and medical complications (Y33: 23) deaths, sharp force injuries (Y28: 22 deaths), electrocutions (Y33: 21 deaths) and transport fatalities of undetermined intent (Y32: 10 deaths).

Approximately half of all injury-related deaths were intentionally inflicted (48.6%; 25 499/52 493). Homicide was the leading apparent manner of death, accounting for 36.2% (19 028/52 493) of all external causes (95% CI: 34.2–38.3) or 38.4 per 100 000 population (95% CI: 33.8–43.0). The male homicide rate (67.4; 95% CI: 58.9–75.8) was significantly higher than the female rate (11.3; 95% CI: 9.5–13.0).The male-to-female ratio of homicide (6 male deaths per female death) was higher than for any other apparent manner of death. This was due to the particularly high rate ratios for three major external causes of death that were attributed to homicide: sharp force injuries/stabbing, gunshot injuries and blunt-force injuries (Table 2).

The suicide rate of 13.4 per 100 000 population (95% CI: 11.6–15.2) was approximately one third of the homicide rate. Males were again over represented and overall there were five male suicides for every female suicide.

Transport-related injuries accounted for more than one-third of all external causes of death 33.8% (17 742/52 493; 95% CI: 31.9–35.7) and the majority of deaths due to unintentional injury. Most of the transport-related deaths were due to road traffic injuries, which represented 32.6% (17 103/52 493) of all injury-related deaths (95% CI: 30.7–34.5) or 36.1 per 100 000 population (95% CI: 30.9–41.3). Pedestrian deaths accounted for 40.0% (5604/14 010) of the road traffic deaths in which the road user group was defined. Drivers and passengers accounted for 22.9% (3205/14 010) and 32.6% (4572/14 010) of deaths respectively (Table 2). The other major causes of unintentional injuries included burns, drowning and falls. The male-to-female mortality ratios were lower for accidental than for intentional injuries.

**Fig. 2:**
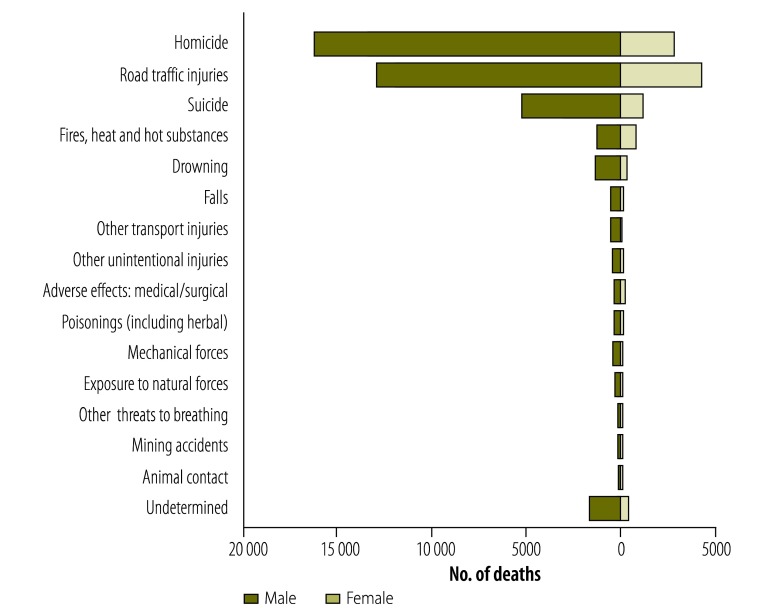
Male and female deaths by injury categories, South Africa, 2009

Note: Weighted analysis, male *n* = 41 807, female *n* = 10 541.

For a small subset of deaths from external causes (4.0%; 2099/52 493) it was not possible for the medical examiners to determine intent. This was most common among deaths arising from the ingestion of poisonous substances (including drugs), deaths from fires, burns and hot substances, and instances where decomposed bodies, bones or skeletons were found. Fig. 2 shows the distribution of male and female fatalities.

Gunshot injuries were a leading cause across several categories, accounting for 6428 deaths, equivalent to 17.6 firearm-related deaths per day (95% CI: 15.7– 19.6). Of these, 5513 were homicides, 780 were suicides, 48 were unintentional and 87 were deaths of undetermined intent.

Table 3 presents the metro and non-metro mortality rates for homicide, suicide and road traffic injuries by sex, age and race. For all injuries and for homicide, metro mortality rates were notably higher than for non-metro areas. This finding was consistent when the data were stratified by sex and age.

**Table 3:** Mortality from homicide, suicide and road-traffic injury for metro and non-metro areas, South Africa, 2009

External cause of death	Metro areas^a^			Non-metro areas^a^			Total^a^		RR
	No. (95% CI)	Mortality rate per 100 000 population (95%CI)^b^		No. (95% CI)	Mortality rate per 100 000 population (95%CI)^b^		Metro/non-metro	Mortality rate per 100 000 population (95%CI)^b^	
**Homicide (X85**–**Y09)**	9 846 (8 328–11 364)	45.1 (37.7–52.5)		9 182 (7 004–11 361)	33.2 (25.2–41.1)		19 028 (16 852–21 204)	38.4 (33.8–43.0)	1.4
Male	8 592 (7 266–9 919)	78.3 (65.2–91.4)		7 652 (5 784–9 521)	58.5 (43.9–73.1)		16 245 (14 339–18 151)	67.4 (58.9–75.8)	1.3
Female	1 232 (1 025–1 440)	12.1 (9.3–15.0)		1 508 (1 184–1 832)	10.6 (7.8–13.4)		2 740 (2 440–3 041)	11.3 (9.5–13.0)	1.1
0–4 years	101 (80–122)	5.3 (4.2–6.4)		185 (143–227)	5.2 (4.0–6.4)		286 (243–328)	5.2 (4.4–6.0)	1.0
5–14 years	109 (78–140)	3.6 (2.6–4.6)		178 (64–291)	2.8 (1.0–4.6)		287 (172–401)	3.1 (1.8–4.3)	1.3
15–29 years	4 401 (3 755–5 046)	69.9 (59.7–80.2)		4 130 (3 074–5 187)	47.2 (35.1–59.3)		8 531 (7 466–9 596)	56.7 (49.6–63.8)	1.5
30–44 years	3 207 (2 730–3 685)	67.3 (57.3–77.3)		2 648 (2 135–3 161)	50.9 (41.1–60.8)		5 855 (5 346–6 365)	58.7 (53.6–63.8)	1.3
45–59 years	1139 (948–1 330)	40.1 (33.3–46.8)		1 156 (896–1 416)	32.6 (25.3–40.0)		2 295 (2043–2 547)	35.9 (32.0–39.9)	1.2
≥ 60 years	403 (299–507)	26.8 (19.9–33.7)		592 (426–758)	24.2 (17.4–31.0)		995 (822–1 169)	25.2 (20.8–29.6)	1.1
Black	8 113 (6 953–9 273)	52.6 (44.6–60.5)		7 974 (5 836–10 113)	33.8 (24.6–43.0)		16 088 (14 011–18 164)	41.2 (35.6–46.9)	1.6
Coloured	1 080 (685–1 474)	45.3 (26.8–63.7)		848 (476–1 219)	40.2 (22.0–58.6)		1 927 (1 395–2 459)	42.9 (29.9–55.9)	1.1
Asian	226 (175–276)	22.0 (14.5–29.6)		91 (54–127)	30.8 (15.1–47.9)		316 (258–375)	24.0 (16.8–31.2)	0.7
White	394 (257–531)	11.3 (6.7–15.9)		226 (167–286)	11.6 (6.5–16.7)		620 (484–756)	11.4 (8.0–14.8)	1.0
**Suicide (X60**–**X84)**	2 982 (2 469–3 496)	14.0 (11.3–16.8)		3 488 (2 772–4 205)	12.9 (9.9–15.9)		6 471 (5 753–7 189)	13.4 (11.6–15.2)	1.1
Male	2 444 (2009–2 878)	23.3 (18.4–28.1)		2 864 (2 285–3 442)	23.2 (17.6–28.8)		5 307 (4 717–5 898)	23.2 (19.9–26.5)	1.0
Female	533 (449–617)	5.0 (3.8–6.2)		620 (439–802)	4.1 (2.7–5.6)		1 153 (976–1 331)	4.5 (3.5–5.4)	1.2
5–14 years	46 (36–55)	1.5 (1.2–1.8)		82 (49–115)	1.3 (0.8–1.8)		127 (94–160)	1.4 (1.0–1.7)	1.2
15–29 years	1 181 (1 001–1 362)	18.8 (15.9–21.6)		1 470 (1 136–1 804)	16.8 (13.0–20.6)		2 652 (2 332–2 971)	17.6 (15.5–19.8)	1.1
30–44 years	1 057 (880–1 233)	22.2 (18.5–25.9)		1 102 (865–1 340)	21.2 (16.6–25.8)		2 159 (1919–2 399)	21.7 (19.3–24.1)	1.0
45–59 years	499 (370–629)	17.6 (13.0–22.1)		557 (446–668)	15.7 (12.6–18.9)		1 056 (910–1 203)	16.5 (14.2–18.8)	1.1
≥ 60 years	142 (101–182)	9.4 (6.7–12.1)		220 (160–279)	9.0 (6.6–11.4)		361 (295–428)	9.2 (7.5–10.8)	1.0
Black	1 847 (1 482–2 213)	11.9 (9.2–14.6)		2 757 (2054–3 460)	11.8 (8.4–15.2)		4 604 (3 897–5 311)	11.9 (9.7–14.1)	1.0
Coloured	313 (238–388)	13.0 (9.6–16.3)		266 (190–342)	12.6 (7.9–17.2)		579 (475–682)	12.8 (9.9–15.6)	1.0
Asian	188 (91–286)	17.7 (7.6–27.8)		48 (17–79)	17.4 (2.1–33.4)		236 (134–338)	17.6 (8.7–26.4)	1.0
White	619 (403–835)	19.0 (11.7–26.3)		415 (301–529)	23.5 (15.1–31.8)		1 034 (806–1 262)	20.5 (15.2–25.9)	0.8
**Road-traffic (V00**–**V89)**	6 875 (5 582–8 167)	34.0 (27.0–41.0)		10 228 (7 925–12 531)	38.0 (29.2–46.8)		17 103 (14 781–19 425)	36.1 (30.9–41.3)	0.9
Male	5 371 (4 370–6 372)	53.7 (42.5–65.0)		7 571 (5 909–9 233)	60.8 (46.9–74.6)		12 942 (11 245–14 639)	57.2 (49.0–65.3)	0.9
Female	1 494 (1 192–1 795)	15.1 (11.2–19.0)		2 641 (1991–3 291)	18.3 (13.2–23.3)		4 135 (3 492–4 778)	16.8 (13.7–20.0)	0.8
0–4 years	288 (216–360)	15.1 (11.3–18.9)		454 (342–565)	12.7 (9.6–15.8)		741 (616–867)	13.5 (11.2–15.8)	1.2
5–14 years	391 (327–454)	12.8 (10.7–14.9)		657 (486–829)	10.5 (7.8–13.2)		1 048 (878–1 218)	11.2 (9.4–13.1)	1.2
15–29 years	2 098 (1 719–2 478)	33.3 (27.3–39.4)		3 355 (2 594–4 116)	38.4 (29.7–47.1)		5 454 (4 696–6 211)	36.3 (31.2–41.3)	0.9
30–44 years	2 188 (1 713–2 664)	45.9 (35.9–55.9)		2 991 (2 312–3 669)	57.5 (44.5–70.6)		5 179 (4 459–5 899)	52.0 (44.7–59.2)	0.8
45–59 years	1081 (872–1 290)	38.0 (30.7–45.4)		1 681 (1 321–2041)	47.4 (37.3–57.6)		2 762 (2 396–3 128)	43.2 (37.5–49.0)	0.8
≥ 60 years	517 (425–610)	34.3 (28.2–40.5)		753 (565–941)	30.8 (23.1–38.5)		1 270 (1 089–1 452)	32.2 (27.6–36.8)	1.1
Black	5 148 (4 116–6 180)	38.9 (29.9–47.9)		8 456 (6 320–10 593)	37.1 (27.4–46.8)		13 604 (11 459–15 749)	37.2 (30.9–43.5)	1.0
Coloured	620 (480–760)	27.9 (19.9–35.9)		607 (490–724)	29.0 (21.9–36.1)		1 227 (1 052–1 402)	28.4 (23.0–33.9)	1.0
Asian	316 (216–416)	31.3 (20.0–42.5)		167 (106–228)	57.3 (27.5–87.9)		483 (370–596)	37.0 (25.7–48.3)	0.5
White	761 (502–1 020)	25.4 (15.7–35.2)		975 (694–1 255)	58.7 (37.7–79.8)		1 736 (1 376–2095)	37.2 (27.7–46.6)	0.4
**All-injuries (V00**–**Y34)**	24 584 (20 642–28 526)	118.5 (99.0–138.1)		27 910 (22 269–33 551)	102.5 (81.8–123.3)		52 493 (46 930–58 057)	109.0 (97.1–121)	1.2
Male	19 999 (16 779–23 219)	193.3 (161.5–225.2)		21 808 (17 436–26 179)	172.5 (137.6–207.4)		41 807 (37 431–46 183)	181.0 (161.3–200.7)	1.1
Female	4 512 (3 755–5 269)	45.2 (36.7–53.8)		6 029 (4 752–7 306)	41.4 (32–50.9)		10 541 (9 306–11 777)	42.7 (37.1–48.4)	1.1
0–4 years	991 (806–1 176)	51.9 (42.2–61.6)		1 256 (992–1 520)	35.1 (27.7–42.5)		2 247 (1963–2 530)	41.0 (35.8–46.1)	1.5
5–14 years	860 (722–998)	28.2 (23.7–32.7)		1 424 (1 029–1 820)	22.7 (16.4–29.0)		2 285 (1901–2 668)	24.5 (20.4–28.6)	1.2
15–29 years	8 871 (7 556–10 186)	140.9 (120.0–161.8)		10 181 (7 990–12 372)	116.4 (91.4–141.5)		19 052 (16 914–21 190)	126.7 (112.5–140.9)	1.2
30–44 years	7 636 (6 363–8 908)	160.1 (133.4–186.8)		7 868 (6 465–9 271)	151.3 (124.3–178.3)		15 503 (14 068–16 939)	155.5 (141.1–169.9)	1.1
45–59 years	3 394 (2 811–3 978)	119.4 (98.9–139.9)		4 193 (3 409–4 978)	118.3 (96.2–140.5)		7 587 (6 822–8 352)	118.8 (106.8–130.8)	1.0
≥ 60 years	1 620 (1 352–1 887)	107.6 (89.8–125.3)		2 096 (1 623–2 569)	85.8 (66.5–105.2)		3 716 (3 260–4 172)	94.1 (82.6–105.7)	1.3
Black	18 924 (15 826–22 022)	132.8 (109.2–156.4)		23 335 (17 859–28 812)	100.6 (76.7–124.4)		42 259 (36 893–47 625)	112.1 (97.1–127.0)	1.3
Coloured	2 414 (1 762–3 065)	105.5 (76.0–135.0)		2 246 (1 569–2 923)	107.6 (75.4–139.9)		4 660 (3 747–5 573)	106.5 (84.9–128.1)	1.0
Asian	878 (587–1 169)	86.5 (54.9–118.0)		333 (201–465)	115.0 (58.5–172.2)		1 211 (901–1 521)	92.8 (65.4–120.2)	0.8
White	2 236 (1 535–2 936)	69.9 (46.6–93.1)		1 877 (1 376–2 377)	108.0 (74.9–141.0)		4 112 (3 319–4 905)	83.3 (65.4–101.1)	0.6

CI: confidence interval; RR: rate ratio.

^a^ For metro area: unweighted *n* = 12 037 and weighted  *n* = 21 568. For non-metro areas: unweighted *n* = 12 160 and weighted  *n* = 30 925. For total: unweighted *n* =  24 197 and weighted  *n* = 52 493.

^b^ Age-standardized to the WHO world standard population.

Note: The categories for race are those used in postmortem and police reports in South Africa.

Male injury rates were consistently and significantly higher than female rates in both metro and non-metro areas, with the highest male-to-female mortality ratio presenting among metro homicides (7 male deaths for every female death) and non-metro suicides (4.6 male deaths for every female). For road traffic fatalities, there were 3.6 male deaths for every female.

The age pattern for homicide was similar across metro and non-metro areas, albeit with the metro rates being noticeably higher in all but the youngest age category. Homicide rates were highest among teenagers and young adults in the 15–29 years age group in metro areas, and the 30–44 years age group in non-metro areas. Within age strata, the differences between metro and non-metro areas were not significant except for teenagers and young adults (15–29 years age group) among whom metro homicide rates were significantly higher, subjecting them to twice the risk of their non-metro counterparts. Suicide rates by age followed a similar pattern to homicide rates.

**Fig. 3:**
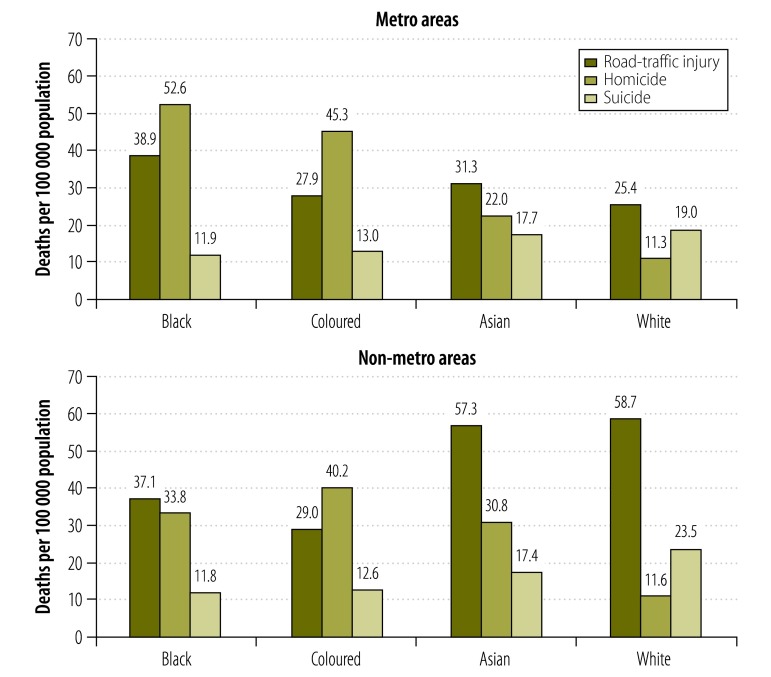
Homicide, suicide and road-traffic injury mortality rates by race in metro and non-metro areas, South Africa, 2009

Note: Weighted analysis, male *n* = 42 602. The categories for race are those used in postmortem and police reports in South Africa.

Fig. 3 shows the homicide, suicide and road-traffic injury rates by race for metro and non-metro areas. The metro and non-metro homicide and suicide patterns by race were inconsistent. People categorized as coloured experienced the highest homicide risk overall. Homicide rates among people categorized as black were highest in metro areas; conversely, among people categorized as white, the highest rates were found in non-metro areas. 

Road traffic mortality rates were higher in non-metro areas for people categorized as black, children and the elderly.

**Fig. 4:**
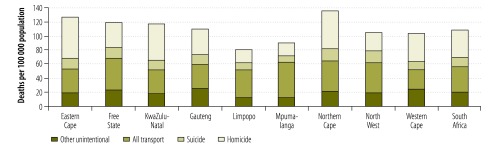
Injury-related mortality rates by province, South Africa, 2009

Note: Weighted analysis, *n* = 42 602.

Differences in metro/non-metro mortality risk affected the provincial mortality profiles depicted in Fig. 4. Homicide ranked highest for five provinces: Eastern Cape, Gauteng, KwaZulu-Natal, the Northern Cape and the Western Cape, of which all except the Northern Cape have large urban centres.

## Discussion

This study provides a comprehensive profile of injury-related mortality in South Africa in the year 2009 and provides cause-specific rates that are not available from other sources. Vital registration data suggest that there has been an overall decrease in external causes of death from 1997 to 2009,^14^^–^^16^ but the reasons are unclear. In those data, a high proportion of deaths were recorded as “other external causes” of accidental injury – 63% (31 166/49 456) in 2009.^15^ The ICD-10 convention to code injury deaths with limited information on intent as accidental (X59) is a common cause of information loss for injury-related mortality data internationally.^17^

We found more than three times as many deaths from homicide and road-traffic injury than were recorded by vital registration.^15^ Underreporting was also apparent in other official statistics. We recorded significantly more homicides (13% higher) than the 16 834 recorded by the South African Police Service in 2009 and significantly more road deaths (24% higher) than the 13 802 recorded by the Road Traffic Management Corporation.^18^^,^^19^

The estimated total number of injury-related deaths in our study did not differ significantly from the 49 456 deaths from external causes recorded in vital registration,^15^ but better cause-specific detail is required for modelling of burden of disease trends. Previously, the first South African burden of disease study^2^ provided the only detailed national estimates by age and sex for major causes of injury. There has been an overall decrease in total injury-related deaths from an estimated 59 935 in the year 2000.^1^^,^^2^ The homicide rate of 38.4 per 100 000 population still places South Africa among the most violent countries, but there has been a significant decrease since 2000 when the national homicide rate was estimated at 64.8 per 100 000 population. This is consistent with national police statistics^18^ and retrospective national surveys of female homicides that also indicate a decrease.^9^ According to police statistics, homicide decreased from 18 793 to 15 609 between 2004 and 2011^18^. Two nationally representative retrospective surveys measured a 38% decrease (3793 to 2363 deaths) in female homicide between 1999 and 2009.^9^

The decrease in female homicide has been attributed partly to the effectiveness of the Firearms Control Act of 2000.^9^ A recent analysis of homicide across five South African cities from 2001 to 2005 confirms a substantial year-on-year decrease in homicides involving firearms, coinciding with the implementation of the Firearms Control Act, alongside a more modest decrease in other means of homicide.^20^ We estimate that homicides involving firearms in metro areas accounted for just 38.5% of homicides in 2009, compared to 44% in 2005.^20^ This suggests that homicide involving firearms has declined more rapidly than homicide by other means, at least in urban areas. The exceptionally high homicide rate among males has been noted previously,^2^ but the male-to-female ratio has increased since 2000 indicating that the decrease in male homicide has not kept pace with the greater decline among females. After taking into account the overall decrease, the pattern of homicides by age group was similar to that in the year 2000.

Suicide remains an important contributor to injury-related mortality, although our study does suggest a decrease in the female suicide rate from an estimated 6.1 per 100 000 population in 2000 to 4.5 per 100 000 population in 2009. Analysis by age shows a slight decrease, compared to 2000 estimates, among adults younger than 44 years, which is offset by an increase among older adults. As has been shown previously,^21^ higher suicide rates were associated with increasing socioeconomic status, which was consistent across metro and non-metro areas.

Traffic authorities reported a slight increase in deaths related to road traffic injuries.^22^ In contrast, a recent global study reported a consistent rate for road traffic mortality in South Africa from 2000 to 2010.^23^ We found that deaths from road-traffic injury have not decreased significantly since the year 2000. If the homicide rate continues to decline, deaths from road-traffic injury are on course to become the leading cause of injury-related mortality. Road traffic injuries are the largest contributor to injury-related mortality in three of South Africa’s predominantly rural provinces: Limpopo, Mpumalanga and the North West Province. At a national level, the prominence of pedestrian fatalities is of particular concern as it suggests that the strategy to improve road safety has not met the needs of vulnerable road users. Pedestrian safety relies on reduced exposure to risk through improved safety infrastructure and the provision of alternative transport modes for vulnerable road users, as behavioural modifications to reduce the risk of crash involvement and severity in the event of a collision are not easily attained.^24^

This study presents a national profile of injury-related mortality by race, which in South Africa provides a rough proxy for socioeconomic status. Contrary to the conventional discourse that violence is more concentrated in areas of poverty and deprivation,^25^^,^^26^ our study reveals that peopled categorized as coloured (who are, on average, more affluent than black people),^27^ have comparable rates of homicide to black people. This is due to the relatively high rates of homicide among people categorized as coloured in non-metro areas, especially in the Western and Northern Cape. These two provinces have high levels of violence and of alcohol-related harm. Metro homicide rates were higher among racial categories that are, on average, poorer.^20^

Comparison of our findings with estimates^2^ for the year 2000 highlights important changes in the profile of deaths from external causes. Closer inspection reveals potential inaccuracies from the modelling process and the triangulation of inherently limited data sources that may have affected the earlier estimates. A previous study recognized the urban bias of mortuary-based surveillance data^2^ as well as the poor distinction between deaths of undetermined cause (i.e. whether from natural or external causes) and injury-related deaths of undetermined intent. Our data confirm the incompleteness and misclassification of vital registration data from the Department of Home Affairs, which codes as R99 (i.e. an ill-defined natural cause death) any death that is under investigation at the time of certification.

 The homicide rate in our study was 5% higher than the 36.4 per 100 000 population in the global burden of disease study,^23^ while mortality from road-traffic injury and suicide were approximately fourfold higher than the 8.9 and 3.6 per 100 000, respectively, in the global burden of disease study. We have demonstrated the feasibility and utility of using mortuary-based data to provide timely, accurate and representative injury-related mortality information to monitor major injury trends and to identify at-risk groups.

The study provides empirical evidence of the extent of misclassification and underreporting that compromises the evaluation of violence and injury prevention efforts. Comparison with several official sources and secondary analyses that rely on these sources suggests that mortuary data can improve estimates of mortality from external causes, and complement national and global burden of disease estimates.
